# Inactive Hepatitis B Carrier and Pregnancy Outcomes: A Systematic Review and Meta–analysis

**Published:** 2017-04

**Authors:** Afsaneh KERAMAT, Masud YOUNESIAN, Mohammad GHOLAMI FESHARAKI, Maryam HASANI, Samaneh MIRZAEI, Elham EBRAHIMI, Seyed Moaed ALAVIAN, Fatemeh MOHAMMADI

**Affiliations:** 1. Dept. of Reproductive Health, Student Research Committee, School of Nursing and Midwifery, Shahroud University of Medical Sciences, Shahroud, Iran; 2. Dept. of Environmental Health Engineering, School of Public Health, Tehran University of Medical Sciences, Tehran, Iran; 3. Dept. of Biostatistics, Faculty of Medical Sciences, Tarbiat Modares University, Tehran, Iran; 4. Liver and Pancreatobiliary Diseases Research Center, Digestive Disease Research Institute, Shariati Hospital, Tehran University of Medical Sciences, Tehran, Iran; 5. Research Center for Gastroenterology & Liver Diseases, Baqiyatallah University of Medical Sciences, Tehran, Iran

**Keywords:** Inactive hepatitis B, Pregnancy outcome, Pregnancy adverse effect

## Abstract

**Background:**

We aimed to explore whether maternal asymptomatic hepatitis B (HB) infection effects on pre-term rupture of membranous (PROM), stillbirth, preeclampsia, eclampsia, gestational hypertension, or antepartum hemorrhage.

**Methods:**

We searched the PubMed, Scopus, and ISI web of science from 1990 to Feb 2015. In addition, electronic literature searches supplemented by searching the gray literature (e.g., conference abstracts thesis and the result of technical reports) and scanning the reference lists of included studies and relevant systematic reviews. We explored statistical heterogeneity using the, I2 and tau-squared (Tau2) statistical tests.

**Results:**

Eighteen studies were included. Preterm rupture of membranous (PROM), stillbirth, preeclampsia, eclampsia, gestational hypertension and antepartum hemorrhage were considerable outcomes in this survey. The results showed no significant association between inactive HB and these complications in pregnancy. The small amounts of *P*-value and chi-square and large amount of I2 suggested the probable heterogeneity in this part, which we tried to modify with statistical methods such as subgroup analysis.

**Conclusion:**

Inactive HB infection did not increase the risk of adversely mentioned outcomes in this study. Further, well-designed studies should be performed to confirm the results.

## Introduction

Today, viral hepatitis has a heavy burden on health systems (1). The hepatitis B virus (HBV) as a type of hepatitis viruses can lead to morbidity and mortality on the world (2). The inactive HBV is 100 times (100×) more contagious and more robust than HIV, and it often goes undetected (3). Approximately 25% of sexual contacts of HB infected persons maybe contagious for HBV. HBV is most commonly spread by an infected mother to her infant at birth (4), making it an important issue in the area of pregnancy and childbearing, as it can lead to chronic disease or hepatocellular carcinoma in adults who acquired the virus at birth. “Despite the hepatitis B immunization, HBV infection still is a threatened medical and financial issue and affects young adults in the world. Virus transmission and the fetus health are both the important concerns of women in the world” (5, 6).

As with many diseases in pregnancy, little is known about HBV infection and it was generally accepted that inactive hepatitis B (IHB) infection did not affect gestation or pregnancy outcome (7, 8). However, although conflicting, challenge this belief (9) as some studies have explored the impact of this infection on pregnancy outcomes such as preterm birth, prepartum hemorrhage, gestational diabetes mellitus (GDM, fetal macro-somia or poor the other pregnancy outcomes) (10–30).

Since, awareness of HB effects on pregnancy outcomes is important for both patients and healthcare providers and the study results are controversial we have done this research to partly clarify the effects.

## Methods

### Search Strategy and selection criteria

We used a specific methodology to do this study based on our previous research (17). We searched PubMed, Scopus, and ISI web of science from 1990 to Feb 2015. After the initial assessment of the electronic sources, references identified as potentially relevant examined to identify the three key journals. In addition, electronic literature searches supplemented by searching the gray literature (e.g., conference abstracts thesis and the result of technical reports) and scanning the reference lists of included studies and relevant systematic reviews. We applied a free keywords or MESH words search with the following terms: inactive hepatitis B, “Hepatitis B surface antigen”, Hbs Ag, hepatitis B carrier, pregnancy outcome, prenatal outcome, obstetric outcomes, pregnancy adverse effect, preterm rupture of membranous (PROM), stillbirth, preeclampsia, eclampsia, gestational hypertension, and antepartum hemorrhage.

We included any cohort, case-control, and cross-sectional studies if they had a healthy control group and reported one or more considered maternal or prinatal outcomes. Patients meeting the following criteria were included:

1. Pregnant women previously diagnosed as IHB carriers or diagnosed in routine antenatal blood test in pregnancy;
2. Pregnant women with normal singleton pregnancy with no history of disease or medication consumption.

Studies were excluded if (a) there was no control group of natural conception, (b) obstetric and perinatal outcomes were not reported, (c) the study subjects of primary articles did not have normal singleton pregnancy, or being addicted, or (d) subjects indicated superinfection with hepatitis A, C, D or E virus.

**Fig. 1:**
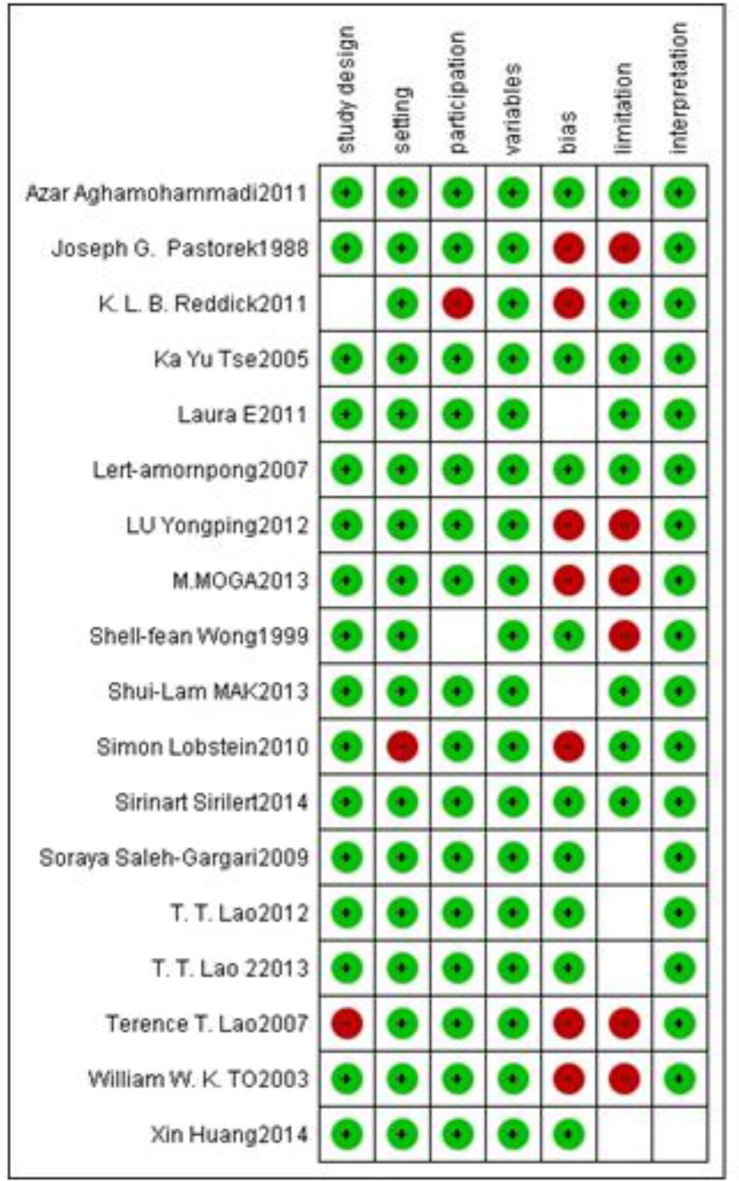
Risk of bias summary: review authors’ judgments about each risk

For quality assessment, we used the modified version of STROBE that includes seven items of STROBE checklist (setting, participation, variables, bias, limitation, and interpretation). There were five low-risk studies and nine high-risk studies among the included studies (Fig. 1). For publication bias assessment, we used the Begg and the Egger tests.

## Results

**Fig. 2:**
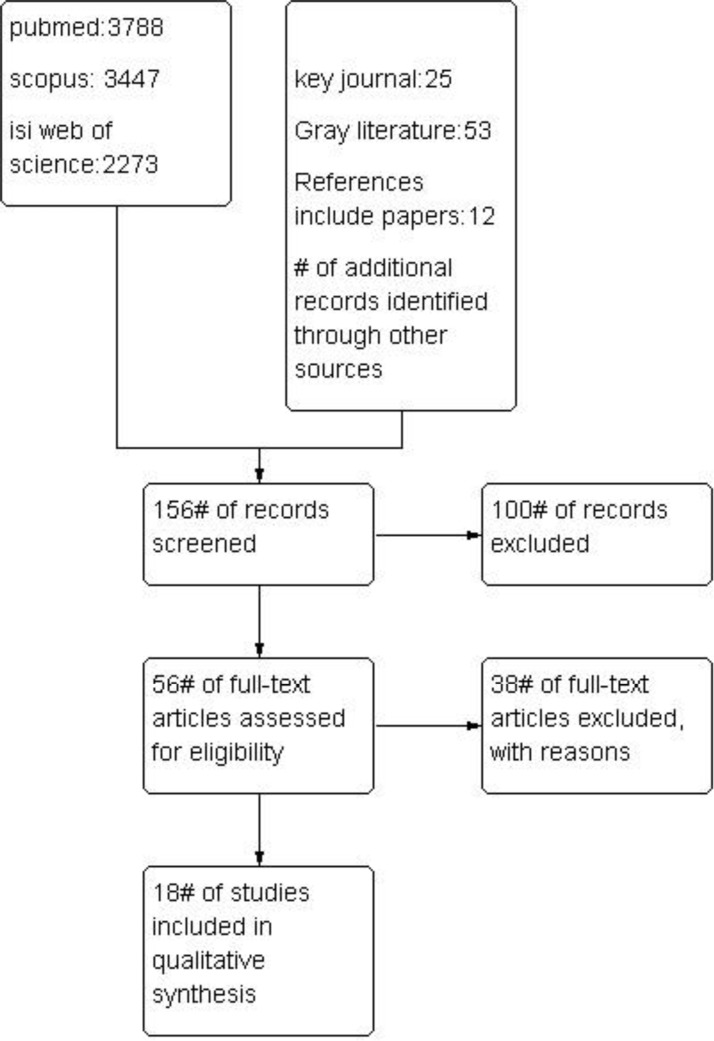
Flow diagram of the progress through the phases of meta-analysis

The results of the literature search are summarized in Fig. 2.

Overall, 156 studies were identified and reviewed by two reviewers. Fifty-six studies were selected for a detailed review, and finally, eighteen studies could include in the meta-analysis. Among the included studies, the outcomes were as follows: Eight studies in preterm rupture of membranous (PROM), six in stillbirth, fourteen in preeclampsia, six in eclampsia, eight in gestational hypertension, and nine in antepartum hemorrhage.

### Meta-analysis of preterm rupture of membranous (prom) and inactive Hepatitis B

Eight of the eighteen studies compared the PROM between the IHB carrier and healthy pregnant women groups (8, 15, 20, 22, 23, 29, 30).

The *P*-value was 0.009 and the corresponding I2 statistic was 63%, suggesting moderate variability among the studies. The pooled OR of having preterm rupture of membranous among inactive HB carrier women versus non-infected women was 1.33 (95% CI, 0.94–1.89). These findings suggest no significant association between inactive HB carrier infection and PROM. Subgroup analysis based on study design (case-control and cohort) could not decrease I2, but subgroup analysis based on quality of papers (high vs. low) could decrease I2 only in low-quality group of study.

### Meta-analysis of stillbirth and inactive Hepatitis B

Six of the eighteen studies compared the stillbirth between the IHB and healthy pregnant women groups (8, 14, 15, 24, 31, 32).

The *P*-value was 0.08 and the corresponding I2 statistic was 50%, suggesting low variability among the studies. The combined OR was 0.9. (95% CI: 0.52–1.55). This effect size measure means that the difference between the two groups (infected vs. non-infected) was not statistically significant.

### Meta-analysis of preeclampsia and inactive Hepatitis B

Fourteen of the eighteen studies compared the preeclampsia between the IHB carrier and healthy pregnant women groups (7, 8, 12–15, 20–22, 24, 29, 31–33). The *P*-value was 1.01 and the corresponding I2 statistic was 71%, suggesting severe variability among the studies. The risk of preeclampsia was similar between the two groups (OR=1.03, 95% CI: 0.78–1.35;. For heterogeneity, we conducted two subgroup analyses based on study design and quality that two analyses could decrease I2 only in cohort study group and low-quality study group, respectively

### Meta-analysis of eclampsia and inactive hepatitis B

Six of the eighteen studies compared eclampsia between the IHB and healthy pregnant women groups (8, 20–22, 24, 31). The *P*-value was 0.9 and the corresponding I2 statistic was 0%, suggesting no variability among the studies. The rate of eclampsia was similar between the two groups (OR = 1.33, 95% CI: 0.49–3.63).

### Meta-analysis of gestational hypertension and inactive hepatitis B

Eight of the eighteen studies compared gestational hypertension between the IHB and healthy pregnant women groups (8, 14, 20–22, 31, 30). The *P-*value was 0.1 and the corresponding I2 statistic was 40%, suggesting low variability among the studies. The rate of gestational hypertension was similar between the two groups (OR=0.9, 95% CI: 0.74–1.08,

### Meta-analysis of antepartum hemorrhage and inactive hepatitis B

Nine of the eighteen studies compared the antepartum hemorrhage between the IHB and healthy pregnant women groups (8, 12–15, 21, 23, 31, 29). The *P*-value was 0.0002 and the corresponding I2 statistic was 74%, suggesting moderate variability among the studies. The two groups have a similar chance for antepartum hemorrhage (OR=1.13, 95% CI: 0.84–1.51). Subgroup analysis based on study quality could decrease I2 in both subgroups.

## Discussion

IHB was not associated with higher rate of adverse pregnancy outcomes such as PROM, stillbirth, preeclampsia, eclampsia, gestational hypertension, ante partum hemorrhage. However, we must pay attention to the amounts of statistical tests of heterogeneity in our interpretation. If we have a meta-analysis with low sample size, the Chi2 test can detect the small amount of heterogeneity that may be clinically unimportant, as was the case in our review. The other parts of heterogeneity can be related to remarkable differences between the study’s results. Moreover, in our study despite the observed heterogeneity, the amount of Tau2 statistic was small and equal to 0.15. This paradox happens when the variance between studies is low and within studies variance is high (34, 35).

Despite the reasons discussed above, where the amount of I2 test was high, we examined the source of heterogeneity with the special methods. In the section related to PROM, heterogeneity was moderate (I2=63%). Subgroup analysis based on quality of studies could decrease the amount of I2 in low-quality studies. A closer look at the effect size estimated by studies in high-quality group showed that low sample size studies had the larger effect size.

In examining the relationship between stillbirth and IHB, analysis results showed IHB infection is not equal to a significant increase in the risk of stillbirth in patients.

A positive effect of IHB on stillbirth may occur because of a higher probability of these pregnancies being accompanied by premature labor, high blood pressure, gestational diabetes, or premature rupture of membranes (15, 24). However, other studies denied this effect and stated, “so that the risk of transplacental infection is low, the fetal risk should not increase”. (8, 14, 31, 32).

Besides, in this part, the amount of I2 indicated that low heterogeneity was observed among the studies. The forest plot showed that the high heterogeneity (15, 24), which had an OR of almost four to nine times that of the other studies. These two studies had lower sample sizes than the other studies in this section.

In the sections related to hypertensive disorder in pregnancy, including preeclampsia, eclampsia and gestational hypertension, IHB did not have a significant effect on these disturbances in pregnancy. Studies that verified the probable effects of IHB on hypertensive disorder in pregnant women said that the systemic inflammation caused by the IHB is responsible for these effects (15, 24, 30). However, some other non-effect studies believed this effect could only be observed in some Asian studies that did not pay attention to the compounding factors such as higher virus loads in the investigated cohort or lack of gynecological and neonatal management in affected pregnant women in these areas (7, 8, 12, 13). The conclusion did not end here in this part because the amount of heterogeneity was different in each part. The study results regarding preeclampsia and hepatitis proposed substantial heterogeneity (I2=70%), therefore, we conducted two subgroup analyses based on quality and study design. Quality subgroup analysis modified the amount of I2 only in the low-quality study group (I2=30%), but the other analysis did not have any effect. The high heterogeneity was indicated visual inspection of forest plot (15) that had an OR almost five times of those in the other studies. In this group, most of the studies were conducted in Hong Kong, and one in Iran, so the racial difference may have a role in this heterogeneity.

The last part of this study is allocated to postpartum hemorrhage and its possible association with inactive hepatitis B. The results of individual studies do not confirm this relationship. Three primary studies that found this result related this effect to increases in both the incidence of placenta previa and placental abruption in these pregnancies (29). Heterogeneity between study results was high, and subgroup analyses based on quality modified it in both groups.

### Strength of evidence

This study was one of the few studies that attempted to evaluate the adverse effects of IHB on the outcome of pregnancy.

### Limitations

The first limitation was importing low-quality studies. The reason for this limitation was the small number of studies done in this area. The second limitation was related to the nature of inactive hepatitis. IHB is endemic in some countries, such as China, so there are many studies conducted in this country that did not have an English abstract and use of them needs to be searched in native languages. The other limitation was about the original articles on meta-analysis performed. Some of these articles have reported the association between IHB viral load and considered outcomes in pregnancy.

## Conclusion

IHB carrier status did not increase the risk of adverse effects such as preterm rupture of membranous (PROM), stillbirth, preeclampsia, eclampsia, gestational hypertension, and antepartum hemorrhage during pregnancy. Women who are IHBcarriers can be managed as any other low-risk pregnancies if they do not have other antenatal complications or associated risk factors such as additional diseases in pregnancy. However, these results should be assessed with further well-designed studies.

## Ethical considerations

Ethical issues (Including plagiarism, informed consent, misconduct, data fabrication and/or falsification, double publication and/or submission, redundancy, etc.) have been completely observed by the authors.
